# Experience of emergency department use among persons with a history of adverse childhood experiences

**DOI:** 10.1186/s12913-020-05291-6

**Published:** 2020-05-24

**Authors:** Eva Purkey, Colleen Davison, Meredith MacKenzie, Tracey Beckett, Daniel Korpal, Katherine Soucie, Susan Bartels

**Affiliations:** 1 Queen’s University Department of Family Medicine, 220, Bagot street, Kingston, Ontario K7L 5E9 Canada; 2 Department of Public Health Sciences, Queen’s University, Kingston, Ontario Canada; 3 Street Health Centre, Kingston, Canada; 4 Family Violence and Crisis Team, Department of National Defense, Kingston, Ontario Canada; 5 Department of Emergency Medicine, Western University, London, Ontario Canada; 6 Department of Emergency Medicine, St Michael’s Hospital, Toronto, Ontario Canada; 7 Department of Emergency Medicine, Queen’s University, Kingston, Ontario Canada

**Keywords:** Emergency Medicine, Vulnerable Populations, Adult Survivors of Child Adverse Events

## Abstract

**Background:**

Adverse childhood experiences (ACEs) are associated with increased morbidity and mortality, lower levels of distress tolerance, and greater emotional dysregulation, as well as with increased healthcare utilization. All these factors may lead to an increased use of emergency department (ED) services. Understanding the experience of ED utilization among a group of ED users with high ACE scores, as well as their experiences as viewed through the lens of a trauma and violence informed care (TVIC) framework, could be important to their provision of care.

**Methods:**

This is the qualitative portion of a larger mixed methods study. Twenty-five ED users with high ACE scores completed in depth interviews. Thematic analysis of the interview transcripts was undertaken and directed content analysis was used to examine the transcripts against a TVIC framework.

**Results:**

The majority of participants experienced excellent care although challenges to this experience were faced by many in the areas of registration and triage. Some participants did identify negative experiences of care and stigma when presenting with mental health conditions and pain crises, as did participants who perceived that they were considered “different” (dressed differently, living in poverty, young parents, etc.). Participants were thoughtful about their reasons for seeking ED care including lack of timely access to their family doctor, perceived urgency of their condition, or needs that fell outside the scope of primary care. Participants’ experiences mapped onto a TVIC framework such that their needs and experiences could be framed using a TVIC lens.

**Conclusions:**

While the ED care experience was excellent for most participants, even those with a trauma history, there existed a subset of vulnerable patients for whom the principles of TVIC were not met, and for whom implementation of trauma informed care might have a positive impact on the overall experience of care. Recommendations include training around TVIC for ED leadership, staff and physicians, improved access to semi-urgent primary care, ED patient care plans integrating TVIC principles, and improved support for triage nurses and registration personnel.

## Background

Great attention is paid to frequent users of the emergency department (ED) because of costs involved in their care. Frequent users represent 5% of all ED users, but account for 21-28% of costs [1]. Several studies indicate that frequent ED users are more likely than other ED users to have an identified family doctor or primary care provider [2] highlighting overall increased healthcare utilization with important implications for healthcare delivery and cost. A 2016 meta-analysis looking at frequent users (often defined as >4 visits per year) highlighted characteristics associated with frequent ED use including socioeconomic factors (low educational status, unemployment, low income) and medical factors (substance use, mental health diagnoses, recent hospital admission) [1]. However, none of the studies included in this meta-analysis investigated adverse childhood experiences (ACE) as a risk factor for increased ED use despite research showing a relationship between ACE and ED utilization, particularly for women and those with a history of childhood sexual abuse [3–5].

Adverse childhood experiences as defined in the ACE study [6] have a known lifelong impact on physical, sexual and mental health. People with a high ACE score (positive response to 4 or more of the 10 questions about adverse childhood experiences such as physical, emotional, or sexual abuse, neglect, or household dysfunction) are more likely to have higher burdens of physical and psychological illnesses [6], which may result in increased care seeking behavior, including in the ED. People with high ACE scores have, on average, lower levels of distress tolerance and poorer emotional regulation [7]. This can lead to difficult interactions in healthcare settings, which may have an impact on access to and receipt of appropriate and acceptable patient-centered care. This in turn may impact health outcomes, further exacerbating health inequities among groups affected by historical and current trauma and adversity. As such, this study sought to [1] explore the experience of ED utilization among a group of ED users with high ACE scores, and [2] examine whether their care experience either included a trauma-informed approach or seemed to indicate that one might be beneficial.

## Methods

### Study design

The findings reported are part of a larger mixed methods study in which a convenience sample of patients presenting to the ED and Urgent Care Center (hereafter included in the term “ED”) of Kingston Health Sciences Centre were surveyed to assess their ACE score using the tool developed by Felitti and Anda [6] (see Additional file 1), as well as to explore other demographic and healthcare utilization factors such as age, reason for visit, additional comorbidities, and frequency of ED utilization. Inclusion criteria for the quantitative study were patients 18 years of age or older, presenting to the ED between May and September 2017 with a Canadian Emergency Department Triage and Acuity Scale (CTAS) of 2-5, able to read and write in English, and in stable enough condition to complete the questionnaire. Patients presenting with a CTAS of 1 were excluded due to the high acuity of their presentations and urgent need for medical care. All participants were to be offered a survey by the registration clerk upon presentation. All gave informed written consent to complete the questionnaire and to have their electronic medical record accessed for further information. A subset of participants consented to be contacted later and the twenty-five qualitative interviews presented here were conducted as part of that follow-up. The data from the larger quantitative component of the study is pending publication and available upon request. All components of the study were approved by Queen’s University Health Sciences and Affiliated Hospitals Research Ethics Board.

### Participants

Participants from the larger quantitative ED sample who gave consent to be contacted were invited to participate in a qualitative interview. Inclusion criteria were participants who [1] had consented on the original survey form to be contacted for further information, and [2] had an ACE score of 4 or higher. Eighty-seven people met the inclusion criteria. Data was incomplete for 9 patients and thus the potential group for qualitative recruitment was 78. ACE scores for potential participants ranged from 4 to 10, and frequency of ED utilization in the 12 months prior to the index visit ranged from 1 to 10. Contact was attempted with all 78 patients. Twenty-eight people were successfully contacted, 3 declined to be interviewed, and twenty-five accepted. All twenty-five volunteer patients completed the interviews (9 men and 16 women) which lasted up to 45 minutes. Saturation was reached in that no new themes were emerging by the end of the interview process [8, 9]. Interviewees were provided with 50 dollars compensation for their time.

### Data collection

The in-depth interviews were conducted by one of three researchers (TB, KS, or research assistant MS) using a semi-structured interview guide developed for this study. All researchers were female, one was a trained trauma therapist (TB), and the other two were a resident (KS) and medical student (MS) with interest in emergency medicine and equity-seeking populations respectively. The interviews were conducted either in person at Queen’s University Department of Family Medicine or over the phone. All participants gave written (in-person) or verbal (recorded over the phone) consent. Interviews were audio-recorded and transcribed verbatim. The structure of the interview script allowed for consistency of administration, and interviewers did not deviate greatly from the scripts provided. Themes explored included experiences in the ED, comparison between primary care and ED, and recommendations for improving experience of ED care. No participants reported experiencing emotional distress as a result of the interview process, although referral mechanisms were in place in case this occurred. None of the participants were known to interviewers prior to interviews taking place.

### Data analysis

Both inductive thematic analysis and directed content analysis was conducted [10]. Inductive thematic analysis is a phenomenological approach which starts with raw data and evolves themes or patterns from this data. Directed content analysis can be used where there is existing theory about a phenomenon but this theory is incomplete [10]. In this case, there is theory about ED use among equity seeking populations [1, 11–13], however there is little theory on ED use among persons with high ACE scores. Likewise, there are Trauma and Violence Informed Care (TVIC) frameworks applied within healthcare, but the use of these clinical frameworks in ED settings is uncommon and there is little to no literature discussing their use in the ED context. In our study, we began with a phenomenological approach exploring and reporting emergent themes from the data, followed by directed content analysis which started with the theory (in this case TVIC), and used data to illustrate and to examine this theory as it pertains to practice in the ED.

Interview transcripts were read in their entirety, analyzed and coded using inductive thematic analysis [14] by two independent researchers (EP and MM), both family physicians working with equity-seeking populations who have experience in qualitative research and clinical and research programs focused on ACEs. NVIVO 12 software was used to establish thematic nodes which were then collated into themes individually by each researcher. Coding and themes were compared, discussed and reviewed extensively using investigator triangulation to ensure trustworthiness of findings. As the focus of the project was on the experience of ED use, themes related to experience of health service utilization were prioritized (over, for instance, the medical diagnoses for which people presented to ED). As an example, the nodes of “respectful treatment”, “feeling heard”, “empathy” and “competency” all contributed to the theme of “positive experiences of ED care” As a second step, themes were then superimposed on a TVIC framework using directed content analysis, again with consensus between two researchers (EP and MM), to better understand whether (a) the experiences of participants met the standards of a TVIC approach and (b) whether any negative patient experiences or identified gaps in care could have been mitigated had a TVIC approach been used. Following analysis of the data, themes were presented back to the entire research team, which included two physicians working in ED (KS and SB). Themes were compared and considered in light of these physicians experience of ED function and work to generate discussion and recommendations.

## Results

### Thematic Analysis (Table 1)

#### Positive Experiences of ED care

Overall, many participants described their experiences of ED care as positive. While they experienced anxiety prior to arrival and had critiques about certain aspects of the system, they were very happy with the quality of care and approach of physicians, nurses, and residents. Participants felt that their concerns were heard, that physicians communicated well with them about medical issues and follow up. The majority of participants felt that physicians were competent to provide excellent care. Participants identified communication as one of the most important aspects of their healthcare experience. All noted the importance of feeling heard and valued having an understanding of their health issues. When communication faltered, such as when participants were left waiting inside the ED for long time periods of time without anyone checking on them or updating them, anxiety could be triggered.

**Table 1:** Thematic Analysis

Theme	Participant Quote (note: P18,M,ACE5 indicates participant no.18, male, with an ACE score of 5)
Positive Experiences of ED Care	*You know, like he was like the kind of doctor that you’re looking for. He was very professional. He took responsibility even though it wasn’t his. And, you know, he was like, this is what’s going to happen. You know, I can’t give you any word on what’s going to happen them, but I can guarantee what’s going to happen to you. You know we got you booked for x-ray right now. You know and it was it was really nice because he addressed every problem basically that he could.* (P20,M,ACE10)
	*A positive experience is that I walked out of there feeling confident that my concerns were heard and my specific concerns were addressed. Now were they treated or left untreated – it would have been explained as to what was going on, why and how and where. And what my role was in fostering a remediation – whatever it was that was bothering me. So I mean it wasn’t just, you know, from the point of view of positive – that’s very positive!* (P10,M,ACE5)
	*I’ve had mostly positive experiences where they’ve actually figured out what’s wrong, when I went there. And I’ve found that they’ve listened. Like I’ve only had a couple instances where they haven’t listened.* (P16,F,ACE6)
Stigmatization as a negative experience of ED care	*When she came in, she explained “there’s nothing we’re going to do for you today, and you might as well go home”. And I said “you’re not listening to me. I’m saying that there’s something wrong with my stomach right now, and I’m telling you that I’m not here seeking drugs”. That’s what really upset me. They looked at me and said “she’s a drug seeker, and we’re not going to do anything for her”. And that really upset me.* (P15,F,ACE7)
	*So I’ve mentioned like some mental health stuff in the past. So there seems to be sort of like a stigma too for that. So like when I show up and I’m in like physically in pain or I’m having issues with my breathing – ok no problem - they can see that there’s something wrong. ( … ) But when I’ve gone before dealing with panic attacks, anxiety attacks—um, people look at you funny and they don’t always take you seriously. They just—I don’t know. Like you can just tell. Like the way they look at you or the way that they talk to you. Even if it is not intentional. I don’t know if they’re always aware that their behavior has that effect.* (P3,F,ACE7)
	*So I think that because I am such a young parent - they look at me like I don’t know what I am talking about. They never listen to you. ( … ) Listen to what we say no matter what we look like!* (P6,F,ACE5)
	*Like I don’t out and dress like your average person so I don’t get your regular looks. So they set that as a personality disorder and all of a sudden I’m a drug addict. I can tell – like I can tell by the look on their face and the questions they ask.* (P20,M,ACE10)
	*I just wish there was maybe better or more training and not like necessarily on how to diagnose something like that but how to work with patients who present those issues. ( … ) Maybe like compassion training or something. Like I feel like that should be mandatory or, I don’t know.* (P3,F,ACE7)
Difficulties at Registration and Triage	*When you go into one of the emerg department, when you’re not in a good state mentally, ah, it’s almost like a house on fire. Like it’s very tight and closed in and so you’re very anxious and you’re in a vulnerable state and it gets amplified by the environment. And the, the staff, some of them seem to have an issue working with patients with mental health.* (P3,F,ACE7)
	*Not so much the nurses or the doctors but the triage people always make me feel like I’m wasting their time.* (P1,F,ACE5)
	*And then it’s like – what’s your concern. And it’s obviously a low concern as far as who’s in the Emerg that day. Um I don’t know, it doesn’t feel good and you’re not sure whether you’re going to be taken seriously* (P23,F,ACE7)
Rationale for Seeking Emergency Care	*Um really it just comes down to speed. Um like if I want thoroughness, I go see my family doctor.* (P20M,ACE10)
	*Generally I will go to, like, I will call my family doctor but if it’s not something I think she can handle there because she doesn’t have all the things and resources that the hospital does, then I will go to KGH* (P25,F,ACE7)
	*Generally it’s new problems. Um yeah it’s generally new but if it’s something that’s happened before and I could never get an answer as to why and it reoccurs and sometimes I go to the Emerg – but generally it’s for new things that are scaring me.* (P1,F,ACE5)
	*Like if I can’t get in to see my doctor. I pick up the phone and they say no he’s busy he really can’t see you or can it wait until tonight. There’s an Emergency Clinic tonight between 5 and 8. If it can’t wait then then I’m going into the Emergency.* (P10,M,ACE5)

#### Stigmatization as a negative experience of ED care

Negative experiences of care were predominantly related to experiences of stigmatization and were confined to certain groups. Participants presenting with pain crises felt that they were labelled as “drug seekers” despite their perceived experience of legitimate pain. Those presenting with mental health concerns felt that ED physicians were not equipped to provide them with adequate care and were often dismissive. Young mothers in our study felt that they were not taken seriously; and persons who presented with, or observed others presenting with unconventional appearances (with respect to dress, for example) felt that they experienced stigma as well. Certain patients who did not describe stigmatizing experiences themselves nevertheless observed such behaviors endured by other patients while they were in the ED together. In all, there was a sense that conformity to particular medical or social norms resulted in best available care, and lack of conformity could result in a more challenging experience.

#### Difficulties at Registration and Triage

Many participants identified ED triage and registration as challenging and stressful. Participants experienced anxiety, fear, tension, or stress prior to arriving at the ED. They were concerned that something might be seriously wrong with them. Several participants spoke of preparing themselves by planning what they would say or how they would present themselves in order to have their concerns taken seriously. Upon arrival, many participants felt that triage staff lacked empathy and compassion, did not seem happy with their job, appeared overworked, and did not really hear participants’ concerns, a situation which often exacerbated an already heightened emotional state for the participant. This experience extended to many participants, and not only those who experienced the stigma mentioned above.

#### Rationale for seeking emergency care

Participants were thoughtful about their reasons for accessing emergency care, and there was remarkable consistency across participants. Participants consulted the ED for three main reasons: First, access: most participants did not have a family doctor. For those who did, could they access their family doctor based on time of day, day of the week, or availability of appointments? If participants had a good relationship with their family doctor, the family doctor was almost always identified as the preferred choice as someone who knew them well, and who helped them to make informed choices related to their health. However, participant expectations around access were such that an appointment offered in several days was often perceived as too long to wait, leading to an ED presentation instead. Second, participants accessed the ED for conditions they felt could not be managed by their family doctor either due to severity or to the need for particular resources (imaging, suture material, etc.). Third, perceived acuity, severity and speed of change in their condition influenced participants’ decision to present to the ED.

### Directed Content Analysis: Trauma and Violence Informed Care (Table 2)

**Fig. 1:**
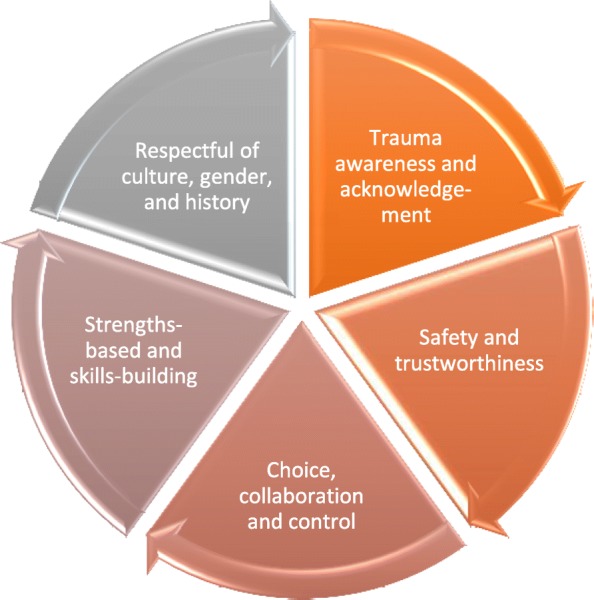
Five Components of a Trauma and Violence Informed Care Approach

Trauma and violence informed care (TVIC) is an approach that takes into consideration the lived experience of past and present trauma and structural violence in patients’ lives. The five components include (1) trauma awareness and acknowledgement; (2) safety and trustworthiness; (3) choice, control and collaboration; (4) strengths based and skills building; and (5) respect for culture, gender and history [15] (see Figure 1). Such an approach is already mainstream in many addictions and mental health services, child welfare services, corrections, and other social service sectors. In conjunction with other components of an equity-oriented care approach, it has been found to improve outcomes in primary care as well [16]. Following thematic analysis, participant narratives were superimposed on a TVIC framework to determine the relevance of such a framework to understanding their experiences of ED care (see Table 2) and gaps in care from a trauma-informed perspective. This was relevant as all participants had a significant historical trauma burden, with an ACE score of 4 or more.

**Table 2:** TVIC Framework directed content analysis

Principles of Trauma and Violence Informed Care	Participant Quotes (note: P18,M,ACE5 indicates participant no.18, male, with an ACE score of 5)
Trauma awareness and acknowledgement	*Heard – no – actually not, never. Absolutely not. (laughter)* *That’s how I feel when I get in there. They know, they think they know everything about my life. Meanwhile not one doctor has ever asked me what I eat or how my bowels work. Not one – ever. (P24,F,ACE7)*
	*I have some unfortunate traumatic experiences having to go to* *Emerg so even personally going there ah it can be triggering to that particular time in my life. I try to avoid that. If I can I’d much rather not do that to myself. (P14,F,ACE7)*
Safety and trustworthiness	*Scared [..] Lonely [..] As if nobody else has ever been in this position (P19,F,ACE9)*
	*Um well when you’re sitting – I don’t – like one of the big issues I find is that you go to triage and then you sit in the waiting room. And sure they may call you in an hour, or whatever to go into the back, but then you sit there for hours sometimes before you see anybody. [ … ] And kind of not see anybody for like an hour, you know, just to check in to say like how’s your pain. Like there’s – I don’t know – there’s no checking in. That’s what really bothers me with Emerg. [ … ] it’s just frustrating as a patient yourself, sitting there waiting and wondering. Is anyone ever going to come and see me? (P15,F,ACE7)*
	*Very tense (P2,M,ACE4)*
Choice, control and collaboration	*Because sometimes doctors will be like yeah shut up you don’t know as much as I do. And they’ll be like, yeah you’re clearly not giving me all the information. Or they’ll try to read around what you’re saying (P20,M,ACE10)*
	*I: Do you ever tell people that you feel they’re not listening to you?* *P: Oh, yes. All the time. I’ll be like “K, well I don’t agree with you, but I guess we have no choice” and they’ll just be like “yup, you’re right. Bye” (P25,F,ACE7)*
	*Like I’m not a doctor and I’m not going to understand your technical terms. But when they break it down to like ok they’re going to do a test. This is kind of what we’re looking for. It will be about 20 minutes. I like that because like it feels like you still have a bit of control as to what’s going on with your health. (P14,F,ACE7)*
	*I think just to basically um to inform patients what’s happening because when you’ve got chest pain and you actually don’t know what’s happening, it’s if someone had come in and said this, this and this is going on. I might have felt better. (P5,F,ACE6)*
Strengths based and skills building	*A positive experience is that I walked out of there feeling confident that my concerns were heard and my specific concerns were addressed. Now were they treated or left untreated – it would have been explained as to what was going on, why and how and where. And what my role was in fostering a remediation – whatever it was that was bothering me. (P10,M,ACE5)*
	*I could see it being a benefit from having some sort of program that would educate or inform when it would be appropriate to go to Emerg or when it would be appropriate to go to a Walk In. When do you feel you need to call 911? I think something along those lines. (P17,M,ACE5)*
	*Um more so confident ah more so like ah just at least knowing that it was most likely to do with my acid reflux. So obviously not medically better until I had the medication in me but I felt I felt better mentally. (P7,M,ACE5)*
Respect for culture, gender and history	*Um mostly just to be respected [ … ] taken seriously, not like um looked down on. Like I don’t out and out dress like your average person so I don’t get your regular looks. [ … ] So every time I go in they’re like – oh you’re an intravenous drug user. I’m like wow that is super fucking insulting. (P20,M,ACE10)*
	*Ahh it’s just that whole non-judgemental, open minded aspect. Um like I said earlier like I don’t need your opinion on my life choices. I don’t need you to tell me that, you know, that people shouldn’t be doing this, this and this. It doesn’t matter because I’ve already done it probably. So either help me or or move on. I don’t – it’s like degrading almost. (P14,F,ACE7)*
	*Like I’ve been to Emerg a lot of time and they’ve given me a prescription for medicine. And they haven’t maybe checked my chart or asked me what my insurance plan was. [ … ] But they’ve given me something that I then could not obtain [ … ]or could not afford. So it’s like – yeah here’s the care you need. And I’ll sit 7 hours to find out I can’t afford this medicine. (P20,M,ACE10)*

Interview questions explicitly and intentionally did not explore participants’ experience of trauma or adversity since this was not the focus of the study, so when these topics arose, it was accidentally through exploration of other themes. Safety and trustworthiness in the context of the ED was conceptualized as timely communication that provided an understanding of what was going to happen next (how long they were going to have to wait, who they would see, what tests would be ordered, etc.). The importance of this was illustrated in our findings by participants’ reported state of mind upon presentation to the ED, which they articulated as anxious, tense, or scared. Participants felt offered choice, control and collaboration if they felt their concerns were heard, if they felt informed about what was wrong with them, and if they were able to engage in a caring dialogue with a healthcare provider. Alternatively, they felt a lack of choice and collaboration when language was dismissive, or when they felt misunderstood or not believed. Participants illustrated a desire for care that was strengths based and skills building by indicating a need to know how to understand and manage their own health, how to access resources that would help them manage health problems in the future, what signs or symptoms to be concerned about, and by seeking out information from pamphlets in the ED informing them about their own health condition. Finally, several participants articulated that care did not respect their history (though details were not sought about gender, ethnicity, or culture of participants) when they experienced stigma or inappropriate care based on their physical appearance, age, income level, or life choices. As highlighted by the quotes in Table 2, participants who did *not* experience care that they felt to be trauma informed perceived these gaps as generally negative.

## Discussion

### Trauma and Violence Informed Care as an Approach to Care Delivery in the context of ACEs as a Determinant of Health

ACEs are a known determinant of adult health. While ACEs are prevalent among the general population [6], we propose that they are a core determinant of health for virtually all equity-seeking groups [17–23] who have documented challenges interfacing with the medical and emergency systems [12, 13, 24–27]. There is also a relationship between ACEs and chronic pain syndromes or mental health concerns [6], both areas which our study highlighted as spaces for improvement in patient care. While current practice appears to be acceptable for many patients with high ACEs, there remains a subset of particularly vulnerable patients for whom additional skills are required to provide accessible and acceptable care. We propose that a core set of enhanced knowledge and competencies on the part of healthcare providers – those articulated in a TVIC framework— could be used in the ED to improve patient care experience.

TVIC can be provided by an individual or at an organizational level, with the latter being preferable as it involves all practitioners and staff as well as the organization of physical space and policy. Providing TVIC does not intrinsically require more time or money, but does require training, consistency, intentionality and awareness on the part of providers and organizational leadership, as well as creativity to restructure new approaches to policy and delivery of care. Studies are emerging examining the effectiveness of this approach on altering health outcomes [16], as well as its use in primary care [16, 28, 29], inpatient mental health [30], emergency departments [31] and non-healthcare settings [32–35]. Literature suggests that service use can be reduced [15, 29], patient comfort and confidence in healthcare improved [16], patient confidence in preventing and managing their own health problems increased [16], and health outcomes improved (eg.: depressive and PTSD symptoms, chronic pain, and quality of life) [16] when people with high ACE scores and other past and current trauma have their needs met through a trauma-informed approach (Fig. 1).

### Clinical Implications and recommendations

Our findings indicate that practitioner-level compassion and communication skills are adequate when interfacing with stable patients with high historical trauma burden who are not suffering from mental health or pain crises and who do not have other characteristics that increase their risk of stigma (poverty, membership in a minority group, substance use, etc). Given what we know about the higher burden of disease and greater healthcare utilization among people with high ACE scores [6], this is positive. Many participants reported experiences in line with the principles of TVIC: they felt heard, they felt safe, they felt that their needs, including the need for communication and understanding, were met.

However, our findings also suggest areas for improvement to better meet the needs of patients with high ACEs and greater dysregulation or emotional instability [7], mental health conditions, substance use or pain disorders, or other experiences of stigma. Our findings further suggest that the principles of TVIC may be helpful for redesigning certain aspects of the care experience as participants identified gaps in services that could be remedied using a TVIC approach. With this in mind, we propose the following:

First, while we recognize that this is outside the purview of the ED, we believe that improving timely access to primary care would decrease the frequency of ED utilization among patients who identify access as their main reason for ED presentation. People with high trauma burden often have low tolerance to distress [7] and a 24 or 48-hour wait to see the family doctor may feel like an unsafe or untenable delay to an individual who is experiencing symptoms as potentially life-threatening, thus leading to increased non-emergent ED utilization. Ensuring that patients can access their family physician team on evenings and weekends, by phone or in person, and that there is availability of same day appointments might increase the likelihood that some patients would either not present to a healthcare professional at all, or that they would present to primary care rather than to the ED, enhancing continuity of care and decreasing more expensive ED utilization.

Second, we propose that all ED staff, from leadership to physicians to triage nurses to registration and janitorial staff, be supported in training around the principles of TVIC. TVIC has been demonstrated as having an impact on health outcomes in primary care, and work is currently being done to study this in the ED context [16, 36]. Enhancing awareness of all team members of the prevalence of ACEs and trauma, as well as the impact that ACEs may have on patients’ presentations, mental health, coping mechanisms and interpersonal interactions, can enhance both provider and patient experience by reducing negative encounters. Small gestures such as giving patients information about how long they can expect to wait for certain aspects of their ED care, checking on them at regular intervals, attending to the impact of neighboring patients, providing printed information, or information on who they are likely to see, when and in which order, can all help to de-escalate patients in states of anxiety or vulnerability. Helping staff to change their perception of patients by considering “what has happened to you?” instead of “what’s wrong with you?”, can help develop empathy, particularly for patients whose concerns seem to fall outside of the traditional, “legitimate” emergent medical complaints.

Third, we propose introduction of enhanced care plans and care coordination integrating the principles of TVIC for frequent ED users, particularly those from equity-seeking groups who are more likely to be met with stigma, or to fear stigma based on previous experiences. This would include focusing on certain patients’ increased need for understanding and reassurance to enhance their sense of safety and autonomy and to decrease likelihood of inappropriate or unnecessary return to care. It would also include providing information on how to manage non-life-threatening conditions (either at home or by accessing primary care) including reasonable wait times for appointments, and serious symptoms warranting return to ED, thereby empowering patients to manage their own health. Involvement of social workers in the early stages of care planning for particularly high users might also help to address underlying mental health or social concerns that may be driving ED utilization. There is some evidence to support the effectiveness of case management in reducing cost and frequency of ED use among high users [37, 38].

Finally, we suggest examining the work-related pressures faced by registration and triage staff in the ED. Supporting staff in these challenging roles might involve decreasing the length of triage or registration shifts, increasing staffing at peak times, adding additional support staff to whom some tasks can be delegated, or adding a physician at triage who may be able to expedite care by ordering labs or imaging ahead of time or by occasionally referring patients to more appropriate services (Table 3).

**Table 3:** Key Points for Emergency Services

1. Most care provided is perceived as excellent by patients.	
2. Certain groups experience stigma, and would benefit from a Trauma and Violence Informed Care approach (TVIC). These groups include: people presenting with emotional instability or dysregulation, people with mental health concerns or substance use disorders, and people with chronic pain syndromes, other traditionally stigmatized groups, among others.	
3. TVIC requires a whole team approach. All people working in an ED from reception and registration to janitorial staff to nurses and physicians should be trained in the principles of TVIC.	
4. TVIC-informed care plans and case coordination should be considered for frequent ED users from equity-seeking groups as this might result in lower ED use.	
5. Attention should be paid to the workload and burnout of registration and triage staff who are the front-line workers in ED.	

### Strengths and Limitations

Our study has limitations related to the sampling methodology of the larger quantitative ED study, resulting in the recruitment of participants who were comparatively more medically and / or emotionally stable. Rather than distributing surveys to all eligible candidates, as per the research protocol, some registration clerks avoided handing out surveys to patients who appeared to be particularly physically or emotionally distressed. As the current qualitative sample was derived from this larger quantitative sample, a bias toward medical and emotional stability at time of presentation was introduced. Given the care concerns highlighted even among the more stable patients that were ultimately included in the study, our results likely under-represent some of the challenges faced by the most vulnerable (and thus most emotionally dysregulated) patients when they seek care in the ED. This hypothesis is also consistent with literature on healthcare experiences among particularly stigmatized groups [39, 40].

## Conclusions

*“I ah I hate hospitals. I absolutely hate them because it always meant that I was being fixed up from a beating eh?”* (P19, Female, ACE Score 9)

Trauma is prevalent in our society. Over two thirds of people have a known history of childhood adversity [6], and this is much higher among equity-seeking populations [17–23]. Ongoing interpersonal and structural violence is a reality for many. Developing the skills and systems required to effectively provide care for people with an experience of trauma is essential for all healthcare providers. Restructuring our systems of care to take into account the lived realities and needs of people with high ACE scores does not require substantial financial or infrastructure investment but does require intention, engagement, and creativity. The focus of this study on the experience of ED utilization among people with high ACE fills a knowledge gap in the ever-growing literature on the impact of ACEs on healthcare utilization. Our study helps to illustrate how a TVIC lens could be applicable and beneficial for ED users. Additional research is underway as further studies are needed to evaluate the impact of TVIC models of care on improving care utilization, patient and provider experience, and patients’ ability to manage their own health.

## Supplementary information

**Additional file 1: Appendix 1**. Adverse Childhood Experiences (ACE) Questionnaire

## Data Availability

The datasets used and analyzed during this study are available from the corresponding author on reasonable request.

## Abbreviations

ACE: Adverse childhood experience
ED: Emergency department
TVIC: Trauma and violence informed care
CTAS: Canadian emergency department triage and acuity scale
