# Supplementary material for: Experience of emergency department use among persons with a history of adverse childhood experiences
Source: BMC Health Serv Res. 2020 May 24;20:455. doi: 10.1186/s12913-020-05291-6 (PMC7245948; doi:10.1186/s12913-020-05291-6)
Supplement: Supplementary file 1 — Additional file 1: Appendix 1. Adverse Childhood Experiences (ACE) Questionnaire [file 12913_2020_5291_MOESM1_ESM.docx]

**Appendix 1: Adverse Childhood Experiences (ACE) Questionnaire**

**** *This is a standardized questionnaire that has been used with thousands of people. We know that the answers to these questions are directly linked to many adult health outcomes*****

**While you were growing up, during your first 18 years of life (circle yes/no):**

1. Did a parent or other adult in the household **often**

Swear at you, insult you, put you down, or humiliate you?

**Or**

Act in a way that made you afraid that you might be physically hurt?

Yes / No

1. Did a parent or other adult in the household **often**

Push, grab, slap, or throw something at you?

**Or**

**Ever** hit you so hard that you had marks or were injured?

Yes / No

1. Did an adult or person at least 5 years older than you **ever**

Touch or fondle you or have you touch their body in a sexual way?

**Or**

Try to or actually have oral, anal, or vaginal sex with you?

Yes / No

1. Did you **often** feel that

No one in your family loved you or thought you were important or special?

**Or**

Your family didn’t look out for each other, feel close to each other, or support each other?

Yes / No

1. Did you **often** feel that

You didn’t have enough to eat, had to wear dirty clothes, and had no one to protect you?

**Or**

Your parents were too drunk or high to take care of you or take you to the doctor if you needed it?

Yes / No

1. Were your parents **ever** separated or divorced?

Yes / No

1. Was your mother or stepmother:

**Often** pushed, grabbed, slapped or had something thrown at her?

**Or**

**Sometimes or often** kicked, bitten, hit with a fist, or hit with something hard?

**Or**

**Ever** repeatedly hit over at least a few minutes or threatened with a gun or a knife?

Yes / No

1. Did you live with anyone who was a problem drinker or alcoholic or who used street drugs?

Yes / No

1. Was a household member depressed or mentally ill or did a household member attempt suicide?

Yes / No

1. Did a household member go to prison?

Yes / No
